# A novel multidrug-resistant cell line from a Chinese patient with pancreatic ductal adenocarcinoma

**DOI:** 10.1038/s41598-024-56464-w

**Published:** 2024-04-22

**Authors:** Huan Tang, Xin Miao, Cheng Yu, Changpeng Chai, Yuanhui Su, Lu Li, Jianfeng Yi, Zhenzhen Ye, Long Miao, Zhengfeng Wang, Hui Zhang, Hao Xu, Wence Zhou

**Affiliations:** 1 The Second Clinical Medical School of Lanzhou University, Lanzhou, 730000 China; 2 The First School of Clinical Medicine, Zhejiang Chinese Medical University, Hangzhou, 310006 China; 3 Department of Anesthesiology, Lanzhou University Second Hospital, Lanzhou, 730000 China; 4 The Fourth Department of General Surgery, the First Hospital of Lanzhou University, Lanzhou, 730000 China; 5 The First Clinical Medical School of Lanzhou University, Lanzhou, 730000 China; 6 The First School of Clinical Medicine of Gansu University of Chinese Medicine, Lanzhou, 730000 China; 7 Department of General Surgery, Lanzhou University Second Hospital, No. 82 Cuiyingmen, Chengguan District, Lanzhou, 730000 Gansu China; 8 Department of Hepatobiliary Surgery, Zhejiang Provincial Hospital of Traditional Chinese Medicine, Hangzhou, 310006 China

**Keywords:** Pancreatic cancer, Cell line, Chemotherapy, Multidrug resistance, Transplanted tumour, Cancer, Cell biology, Gastroenterology, Medical research, Oncology

## Abstract

Chemotherapy resistance poses clinical challenges in pancreatic cancer treatment. Developing cell lines resistant to chemotherapy is crucial for investigating drug resistance mechanisms and identifying alternative treatment pathways. The genetic and biological attributes of pancreatic cancer depend on its aetiology, racial demographics and anatomical origin, underscoring the need for models that comprehensively represent these characteristics. Here, we introduce PDAC-X2, a pancreatic cancer cell line derived from Chinese patients. We conducted a comprehensive analysis encompassing the immune phenotype, biology, genetics, molecular characteristics and tumorigenicity of the cell line. PDAC-X2 cells displayed epithelial morphology and expressed cell markers (CK7 and CK19) alongside other markers (E-cadherin, Vimentin, Ki-67, CEA and CA19-9). The population doubling time averaged around 69 h. In vivo, PDAC-X2 cells consistently maintained their tumorigenicity, achieving a 100% tumour formation rate. Characterised by a predominantly tetraploid karyotype, this cell line exhibited a complex genetic markup. Notably, PDAC-X2 cells demonstrated resistance to multiple drugs, including gemcitabine, paclitaxel, 5-fluorouracil and oxaliplatin. In conclusion, PDAC-X2 presents an invaluable preclinical model. Its utility lies in facilitating the study of drug resistance mechanisms and the exploration of alternative therapeutic approaches aimed at enhancing the prognosis of this tumour type.

## Introduction

Pancreatic cancer ranks among the most malignant tumours globally and stands as the seventh most prevalent cancer, responsible for over 300,000 annual fatalities. Despite advancements in diagnosis and treatment, the disheartening 5-year survival rate hovers between 5 and 10%. Predictions indicate that by 2030, pancreatic cancer might rise to be the second leading cause of tumour-related deaths worldwide^1^. Currently, surgical intervention remains the sole avenue for long-term survival among patients with pancreatic cancer. However, due to the challenge of early detection, most individuals receive diagnoses at advanced stages, leading to a poor overall prognosis. Standard chemotherapeutic options encompass fluorouracil, gemcitabine, platinum, irinotecan, albumin-bound paclitaxel, among others^2–5^. However, the highly heterogeneous and complex tumour microenvironment fosters resistance, complicating treatment strategies^6,7^.

The development of gemcitabine resistance in pancreatic cancer involves a spectrum of mechanisms, including the downregulation of gemcitabine transporters, alterations in gemcitabine catabolism-associated proteins, activation of alternative DNA repair pathways, resistance to apoptosis and promotion of epithelial-mesenchymal transformation (EMT). Moreover, tumours often exhibit cross-resistance, severely limiting treatment therapeutic options^8,9^. Unravelling the molecular underpinning of chemoresistance in pancreatic cancer stands as an urgent need to enhance patient prognoses. To achieve this goal, appropriate preclinical models are indispensable. Considering the diverse origins and ethnic-related genetic variations contributing to pancreatic cancer, employing appropriate preclinical models that encapsulate these characteristics is pivotal^10–13^.

Currently, the majority of cell lines employed in our investigations trace their origins to Western populations (Table 1)^14–18^. The establishment of novel pancreatic cancer cell lines of Chinese origin becomes essential for research purposes.

**Table 1:** Summary of commonly used pancreatic cancer cell lines.

Cell line	Ethnic	Age	Sex	Derived from site	Degree of differentiation	Xenograft formation	Report time
MIA PaCa-2	Caucasian	65	Male	Body and tail	Undifferentiated	Yes	1977^14^
AsPC-1	Caucasian	62	Female	Ascites*	Moderately-differentiated	Yes	1981^15^
PANC-1	Caucasian	56	Male	Head	Undifferentiated carcinoma	Yes	1975^16^
SW1990	Caucasian	56	Male	Tail	Grade II adenocarcinomas	Yes	1983^17^
BxPC-3	Caucasian	61	Female	Body	Moderately-well differentiated	Yes	1986^18^

*The primary lesion is adenocarcinoma of the head of the pancreas.

Here, we introduce a novel pancreatic cancer cell line, PDAC-X2, derived from a Chinese patient. This cell line serves as an in vitro model demonstrating multi-drug resistance, enabling comprehensive studies on resistance mechanisms and the development of innovative therapeutics.

## Materials and methods

### Selection criteria

Inclusion criteria: (1) 18 years < age < 85 years; (2) Diagnosed as malignant tumor through endoscopic biopsy tissue pathology or multiple imaging examinations; (3) Plan to undergo surgical resection or open surgery, laparoscopic and puncture biopsy, or placement of tubes for drainage of malignant pleural and abdominal fluids; (4) Patient informed consent.

Exclusion criteria; (1) Pregnant or lactating women; (2) Suffering from severe mental illness; (3) The patient does not agree to participate.

### Tissue source

**Figure 1:**
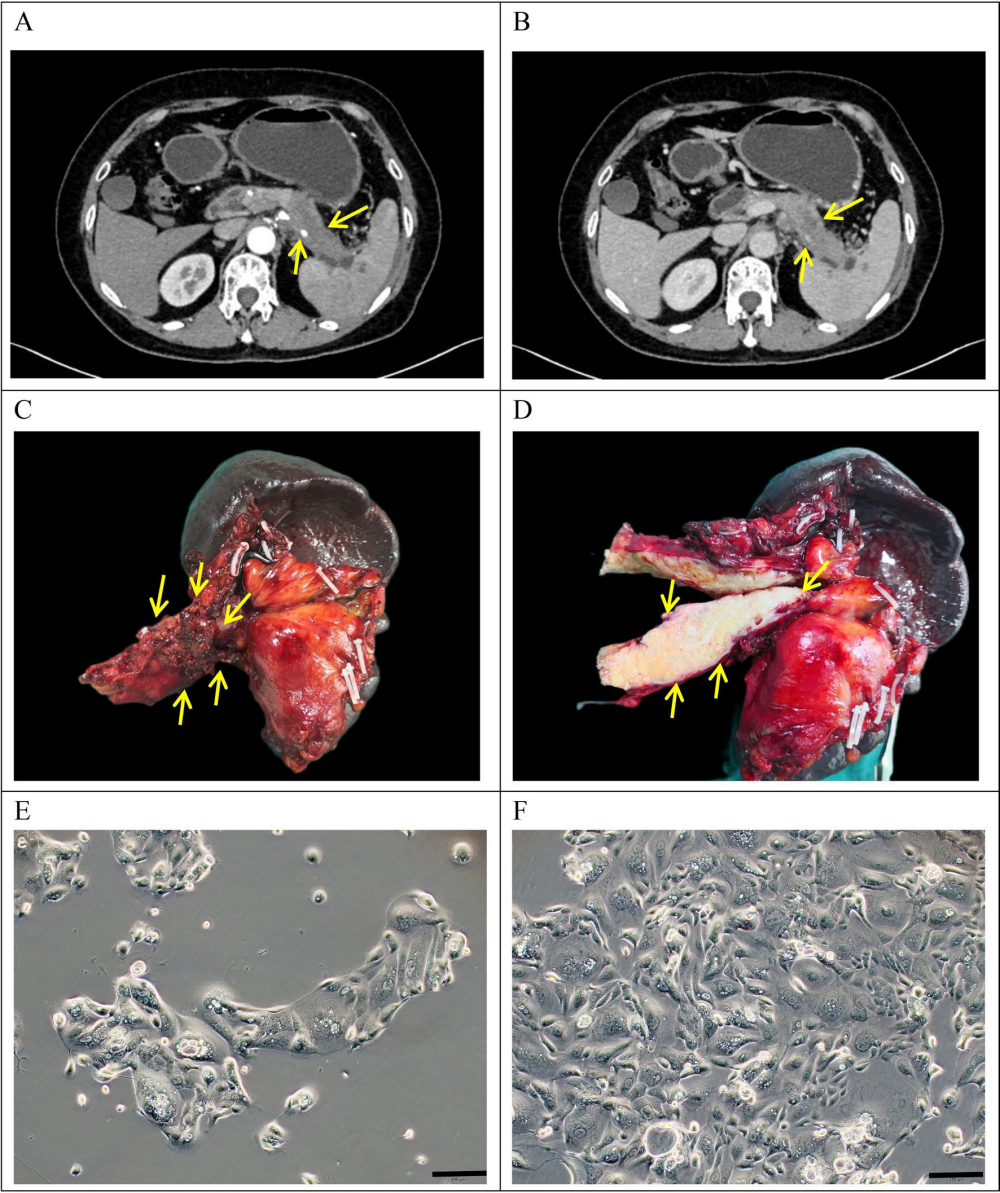
Clinical data and cell morphology. (**A**, **B**) Preoperative computed tomography scan results of the patient depicting a pancreatic tail tumour (indicated by arrows). (**C**, **D**) Gross examination of the postoperative specimen revealed a yellowish–white mass in the pancreatic tail (indicated by arrows). (**E**, **F**) Microscopic examination showing the morphology of PDAC-X2 cells under a light microscope (scale bar = 200 μm).

A 64-year-old female patient, presenting with upper abdominal pain and bloating and with no history of smoking or alcohol consumption, underwent a medical evaluation. Laboratory tests revealed CEA levels at 6.7 ng/ml (reference range 0–5.2 ng/ml), AFP levels at 2.2 U/ml (reference range 0–5.8 U/ml) and CA19-9 levels exceeding 1000 U/ml (reference range 0–35 U/ml) (Table 2). A preoperative CT scan indicated malignant tumours in the pancreas tail, involving the splenic arteries and veins (Fig. 1A,B). The patient did not receive preoperative chemotherapy or radiotherapy. On 4 July 2022, a laparoscopic pancreatectomy with splenectomy was performed at Lanzhou University Second Hospital, retrieving a surgical specimen featuring a 5.5 × 2 cm gray-white, firm tumour at the pancreas tail (Fig. 1C,D). Tissue samples from the primary lesion were isolated to establish a cell line.

**Table 2:** Clinical data of the patient.

Cell line	Patient age/ethicity	Gender	Current Status (days)	Histopathology/differentiation	Tumor size(cm)	Prior therapy	Culture date	Serum		
								AFP IU/ml (0–5.8)	CEA ng/ml (0–5.2)	CA199 U/ml (0–35)
PDAC-X2	64/Asian	Female	Alive	Moderately-poorly	5.5 × 2	None	2022–7-4	2.2	6.7	> 1000

This study received approval from the Medical Ethics Committee of Lanzhou University Second Hospital (2023A-381). The patient provided informed consent.

### NXG mice

Three female NXG mice, aged 5–6 weeks and weighing 11 g and 17 g, were procured from Changzhou Cavens Experimental Animal Co., Ltd. and housed in the SPF-level laboratory of the Animal Experimental Center at Lanzhou University. The animal protocol was designed to minimise pain or discomfort to the animals. The animals underwent a two-week acclimatisation period in laboratory conditions (23 °C, 12 h/12 h light/dark, 50% humidity) with ad libitum access to food and water. The animals were maintained on an autoclaved rodent diet, with bedding, feed and water changed every 2 days. All procedures adhered to institutional and national guidelines for laboratory animal care. Animal health and behaviour were monitored daily for four weeks. Euthanasia was performed by intravenous injection of barbiturate overdose (150 mg/kg pentobarbital sodium) upon reaching a maximum xenograft tumour diameter of approximately1.5 cm or after 1 month of xenograft tumour growth, confirmed by the cessation of heartbeat, breathing and pupil response.

Animal experiments were reviewed and approved by the Medical Animal Experiment Ethics Committee of Lanzhou University Second Hospital (D2023-318). The animal experiment was conducted in accordance with the ARRIVE guidelines and the Guidelines for the Care and Use of Laboratory Animals of China.

The methodologies employed in this study were similar or identical to those reported in previous studies^19,20^.

### Primary culture, cell purification, and cell line establishment

The tumor tissue was washed 3–5 times in sterile phosphate-buffered saline (PBS) (Gibco). Under sterile conditions, it was then finely shredded using a blade and transferred into a mixed solution containing Type II Collagenase (1 mg/ml) (Gibco) and Dispase (Invitrogen) (1 mg/ml) in RPMI-1640 (Gibco), and placed on a shaking table at 37 °C for enzymatic dissociation. The supernatant was sucked out when 1/3rd of the tissue was dissociated, filtered through a 100 mesh filter and centrifuged at 300×*g* for 3 min, and discarded. The precipitate was washed with PBS, centrifuged again, and resuspended in complete culture medium (RPMI-1640 [Gibco] + 10% fetal bovine serum [FBS; VivaCell] + 1% penicillin–streptomycin [BI]). The suspension was then inoculated in a six-well plate (NEST) and cultured at 37 °C under an environment of 5% CO_2_. The culture medium was changed every 48 h. Fibroblasts were removed using a mechanical scraping method. Cell growth was regularly monitored under an optical microscope. When the cells reached 80% conflfluency, they were passaged using 0.25% trypsin digestion. From the third generation onward, cells were passaged at a 1:2 ratio and preserved in serum-free rapid cell cryopreservation solution (Mei5 Biotechnology).

### Analysis of DNA short tandem repeat sequences

PDAC-X2 cells in the logarithmic growth phase (P10) were digested with trypsin, collected after centrifugation and analysed with STR along with the primary tumour tissue by Suzhou Genetic Testing Biotechnology Company to elucidate the correlation between the cells and the primary tumour tissue.

### Cell growth curve

PDAC-X2 cells in the logarithmic growth phase (P20) were digested with 0.25% trypsin, adjusted to a cell density of 1 × 10^4^/ml and inoculated (0.1 ml per well) onto a 96-well plate. After vaccination, CCK-8 (Dojindo) reagent was added simultaneously for four consecutive days, and the UV absorbance at 450 nm wavelength was measured using an enzyme-linked immunosorbent assay reader. The cell doubling time was calculated using the formula Td = t × Lg2/lg (N1/N0), and a cell growth curve was plotted.

### Karyotyping

PDAC-X2 cells (P30) in the logarithmic growth phase were treated with 0.25 μg/mL colchicine for 6 h and incubated overnight at 37 °C. Metaphase cells were then collected and underwent fixation with methanol acetic acid (3:1). After trypsin digestion, specimens were stained with Giemsa staining solution and well-dispersed and moderately stained dividing phases were selected for karyotype analysis under a microscope.

### Scanning electron microscope

Logarithmic growth phase cells (31 passages) were processed for scanning electron microscopy. Cell slides were washed with physiological saline, fixed in 4% glutaraldehyde (SPI-CHEM, USA) and rinsed thrice with phosphate buffer. Subsequently, the slides were dehydrated with 50%, 70%, 80%, 90% and 100% tert-butanol in a step-by-step gradient for 5 min each time. A JEOL JFD-320 cold dryer was used to dry the samples, and the dried samples were taken out after they reached room temperature. A conductive paste was used to coat the sample holder with a JEOL JFCC-160 ion sputterer. Observations and imaging were performed using a HITACHI Regulus 8100 scanning electron microscope after sample preparation.

### Transmission electron microscope

Cells from logarithmically growing PDAC-X2 cells (P31) were centrifuged, fixed in 2% paraformaldehyde–5% glutaraldehyde solution and rinsed with 0.1 mol/L sodium dimethyl arsenate buffer (pH = 7.4). Post-fixation with 1% osmium tetroxide was followed by washing with double distilled water, dehydration in a gradient of ethanol to epoxypropane and embedding with SPI812 resin. Using a Leica EM UC7 slicing mechanism, 1 μm sections were prepared, observed and localised under an optical microscope following azure methylene blue staining. Subsequently, a 60 nm ultra-thin section was prepared using the Leica EM UC7 sectioning mechanism. These sections were stained with uranium acetate and lead citrate, then examined and analysed using a HITACHI H-7650 transmission electron microscope.

### Drug sensitive test

PDAC-X2 cells (P40 and P70), PANC-1 cells and BxPC-3 cells in the logarithmic growth phase were taken, and a single-cell suspension was prepared after enzymatic dissociation. The cell density in the suspension was then adjusted to 1.0 × 10^5^/mL. After thorough mixing, 100 μL of suspension per well was added to a 96-well plate (NEST). After 24 h, anti-tumor drugs (gemcitabine, oxaliplatin, fluorouracil and paclitaxel) were diluted into solutions of different concentrations using complete culture medium, and the culture medium from the 96-well plate was discarded. In the drug treatment group, drug solutions of different concentrations were added to the wells, whereas the control group received complete culture medium. Both groups were set up with four wells each, and 170 μL of drug solution or complete culture medium was added to each well. After 72 h of drug exposure, the drug solution and complete culture medium were discarded from the 96-well plate. A diluted CCK8 solution (10% CCK8 [APExBIO] + 90% RPMI-1640 [Gibco]) at 100 μL was added to each well, along with 100 μL of the diluted CCK8 solution to each of the four blank wells without adding cell suspension. After incubation at 37 °C for 3.5 h, the absorbance at 450 nm was measured using an enzyme-linked immunosorbent assay (BioTek, synergy H1). Drug dose–response curves were plotted and drug IC50 values were calculated using GraphPad Prism 8.0.2 software (GraphPad Inc., San Diego, CA, USA). This analysis was repeated four times.

### Transplantation tumour formation experiment

Logarithmic growth phase cells (P40) were trypsin-digested and adjusted to a cell density of 1 × 10^7^/ml. Cells (0.1 ml per mouse) were inoculated into three NXG mice on the right shoulder and back. Tumour growth in the nude mice was monitored daily, and after four weeks, the mice were euthanised and dissected to observe the growth of the transplanted tumour.

### Immunohistochemical staining

Cells from the 37th generation were grown on sterile slides. After 48 h, slides were washed with PBS, fixed with 4% paraformaldehyde for 15 min, air-dried and treated with 0.5% Triton X-100 for 20 min.

Paraffin sections of transplanted tumours and primary tumour tissues were prepared and baked overnight at 60 °C.

Dewaxing, gradient alcohol hydration and antigen repair were conducted using Dako's Autostainer Link 48 instrument. Blocking peroxidase activity was achieved by incubating with a 3% hydrogen peroxide solution at 37 °C for 15 min. Then, 100 µL of normal goat serum was added dropwise at 37 °C for 15 min. Primary antibodies (CK7, CK19, Ki-67, E-cadherin, Vimentin, CEA and CA19-9) were incubated at 37 °C for 1 h using Fuzhou Maixin ready-to-use antibodies. DAB staining kit (Dako) was utilised for colour development and then rinsed under running water for 5 min. After re-staining with hematoxylin, dehydration and xylene transparency were performed, and neutral resin was used to seal the film for microscopic observation.

### Statistical analysis

All statistical analyses were conducted using SPSS 26.0 software. Data were expressed as mean ± SD. Group comparisons were performed using student’s t-tests and ANOVA. A *P*-value < 0.05 indicated statistical significance.

### Ethical approval

The study was conducted according to the guidelines of the *Declaration of Helsinki* and approved by the Ethics Committee of Lanzhou University Second Hospital (2023A-381). Informed consent was obtained from the patient. The animal procedures were approved by the Medical Animal Experiment Ethics Committee of Lanzhou University Second Hospital (D2023-318).

## Results

### Establishment of PDAC-X2 cell line

Tumour cells isolated from the primary tumour were cultivated under optimal conditions for nine weeks, followed by initial passaging. Approximately a year later, a stable cell line –PDAC-X2– was successfully established. PDAC-X2 cells, observed under an optical microscope, displayed cell adherence and typical epithelial cell-like growth patterns with notable variations in cell size. The presence of multiple megakaryocytes and multinucleated cells with enlarged nuclei and distinct nucleoli, consistent with malignant tumour morphological traits, were observed (Fig. 1E,F). The cell morphology remained consistent despite repeated freezing and resuscitation.

### Analysis of DNA short tandem repeat sequences

**Figure 2:**
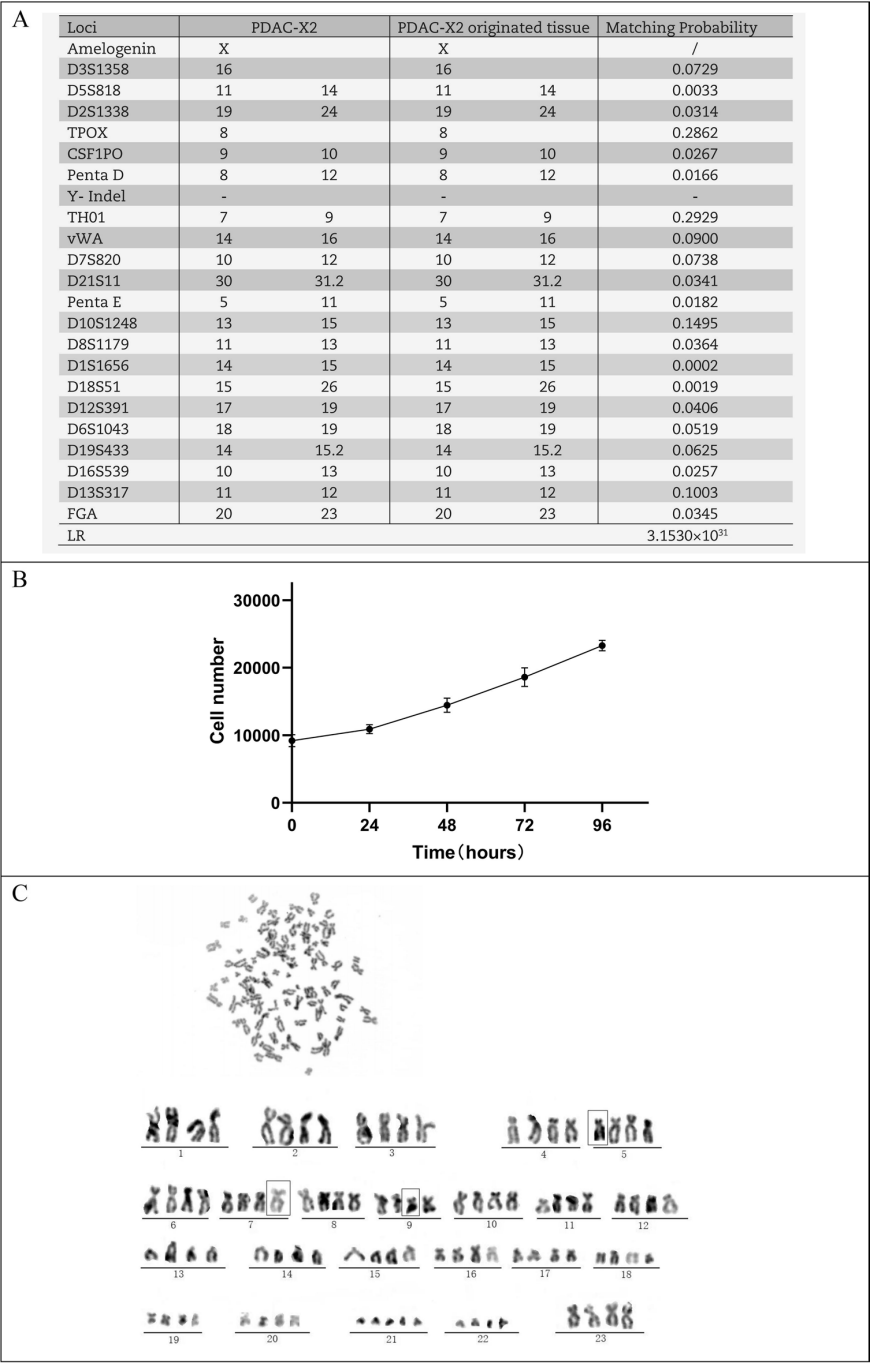
PDAC-X2 cell characteristics. (**A**) Comparative results of short tandem repeat analysis between PDAC-X2 cells and the primary tumour. (**B**) Growth curve illustrating the population doubling time of PDAC-X2 cells. (**C**) Karyotype analysis results for PDAC-X2 cells, with representative karyotype shown as 93, XXXX der (5) der (7) der (9).

DNA typing results indicated a high likelihood (likelihood ratio (LR) = 3.153 × 10^31^ (Fig. 2A)) that PDAC-X2 shares the same origin as the primary tumour tissue. It is an entirely new human pancreatic ductal adenocarcinoma cell not matching any profile in the ExPASy STR database.

### Cell growth curve

PDAC-X2 cells exhibited moderate proliferation rates and a population doubling time of 69 h when cultured in RPMI-1640 culture medium supplemented with 10% fetal bovine serum (Fig. 2B).

### Karyotyping

Karyotype analysis revealed a complex karyotype in PDAC-X2 cells, characterised by significant differences in chromosome number and morphology. Among them, super diploid cells constituted 39%, super tetraploid cells accounted for 44% and sub-heptaploid cells represented 17%. A representative karyotype was identified as 93, XXXX der (5) der (7) der (9) (Fig. 2C).

### Scanning electron microscopy and transmission electron microscopy results

**Figure 3:**
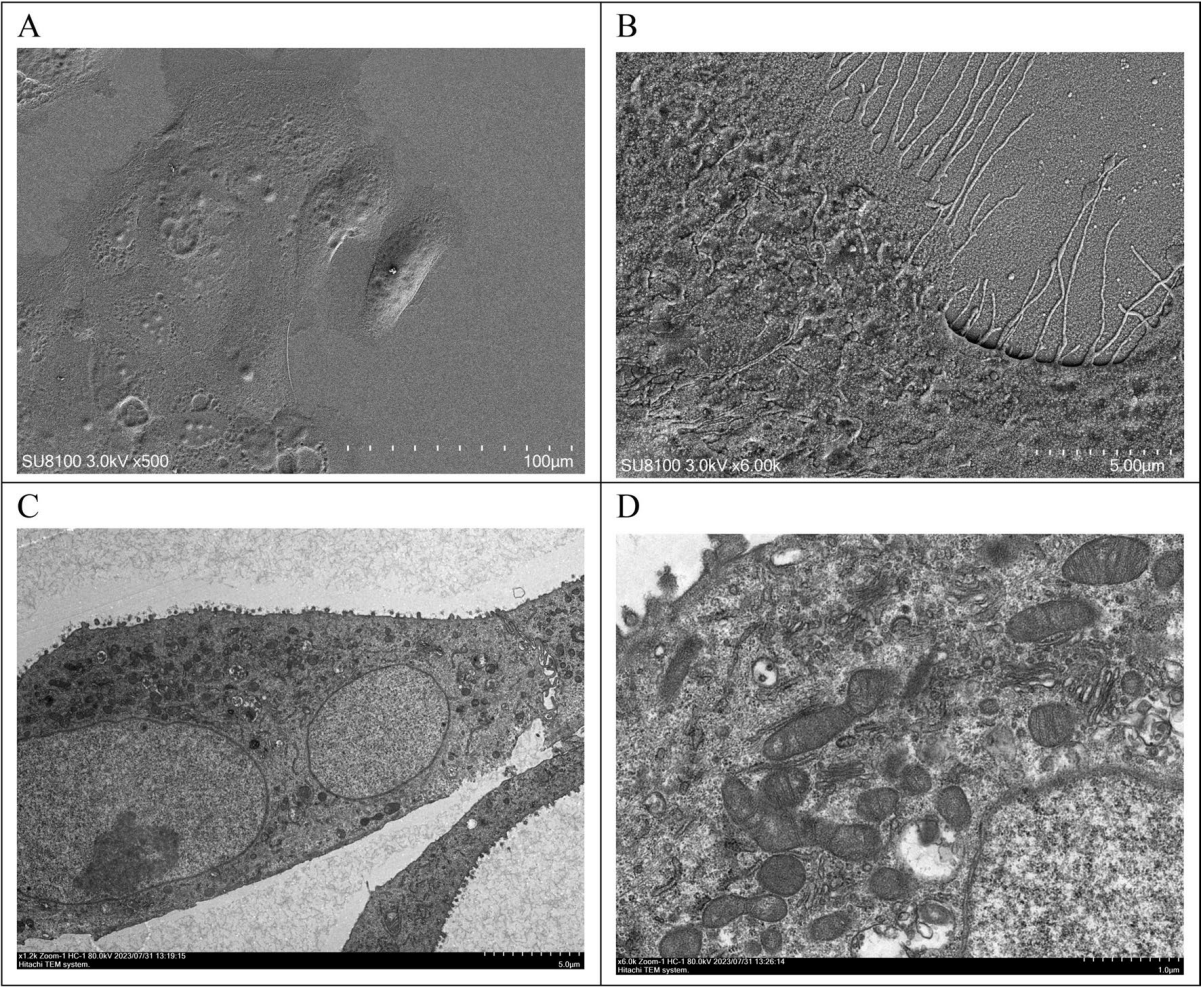
Ultrastructural examination of PDAC-X2 cells using scanning electron microscopy and transmission electron microscopy. (**A**, **B**) Under a scanning electron microscope, PDAC-X2 cells varied in size, with the presence of a small number of microvilli on the cell surface and filamentous pseudopodia. (**C**) Under a transmission electron microscope, the nucleus of PDAC-X2 was large and the nuclear membrane was wrinkled. (**D**) The number of organelles decreased, and mitochondria were significantly swollen and vacuolated along with decreased cristae. Additionally, numerous Golgi apparatus and lysosomes were visible inside the cells.

Scanning electron microscopy revealed diverse-sized PDAC-X2 cells with a limited presence of microvilli on the cell surface and visible filamentous pseudopodia (Fig. 3A,B). Transmission electron microscopy depicted enlarged nuclei with wrinkled nuclear membranes, reduced organelles, significantly swollen and vacuolated mitochondria with decreased cristae, along with increased Golgi apparatus and lysosomes within the cells (Fig. 3C,D).

### Drug sensitive test

**Figure 4:**
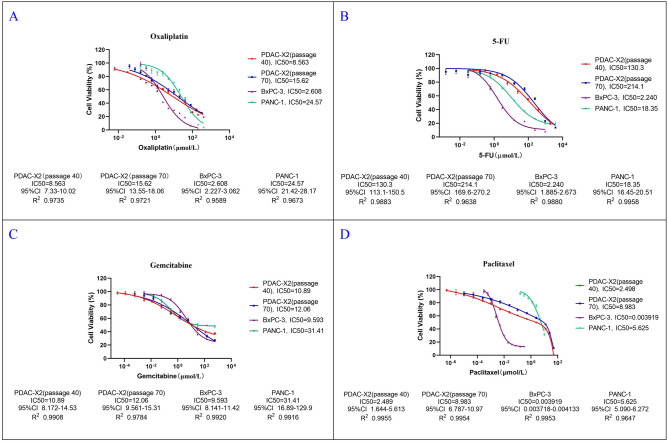
Assessment of anti-cancer drug sensitivity in PDAC-X2 cells (P40 and P70), PANC-1 cells, and BxPC-3 cells. PDAC-X2 cells (P40 and P70) demonstrated resistance to oxaliplatin (**A**), 5-fluorouracil (5-FU) (**B**), gemcitabine (**C**) and paclitaxel (**D**); PANC-1 demonstrated resistance to oxaliplatin (**A**); gemcitabine (**C**); paclitaxel (**D**); and sensitiveness to fluorouracil (**B**). BxPC-3 demonstrated sensitiveness to oxaliplatin (**A**); fluorouracil (B); paclitaxel (**D**); and resistance to gemcitabine (**C**).

PDAC-X2 exhibited resistance to multiple drugs: oxaliplatin (P40, IC50 = 8.56 µmol/L; P70, IC50 = 15.62 µmol/L); fluorouracil (P40, IC50 = 130.3 µmol/L; P70, IC50 = 214.1 µmol/L); gemcitabine (P40, IC50 = 10.89 µmol/L; P70, IC50 = 12.06 µmol/L); paclitaxel (P40, IC50 = 2.498 µmol/L; P70, IC50 = 8.983 µmol/L). PANC-1 was resistant to oxaliplatin (IC50 = 24.57 µmol/L); gemcitabine (IC50 = 31.41 µmol/L); paclitaxel (IC50 = 5.625 µmol/L); and was sensitive to fluorouracil (IC50 = 18.35 µmol/L). BxPC-3 was sensitive to oxaliplatin (IC50 = 2.608 µmol/L); fluorouracil (IC50 = 2.24 µmol/L); paclitaxel (IC50 = 0.003919 µmol/L); and was resistant to gemcitabine (IC50 = 9.593 µmol/L) (Fig. 4A–D). This characterised PDAC-X2 as an intrinsic multidrug-resistant pancreatic cancer cell line.

### Transplantation tumour formation experiment

**Figure 5:**
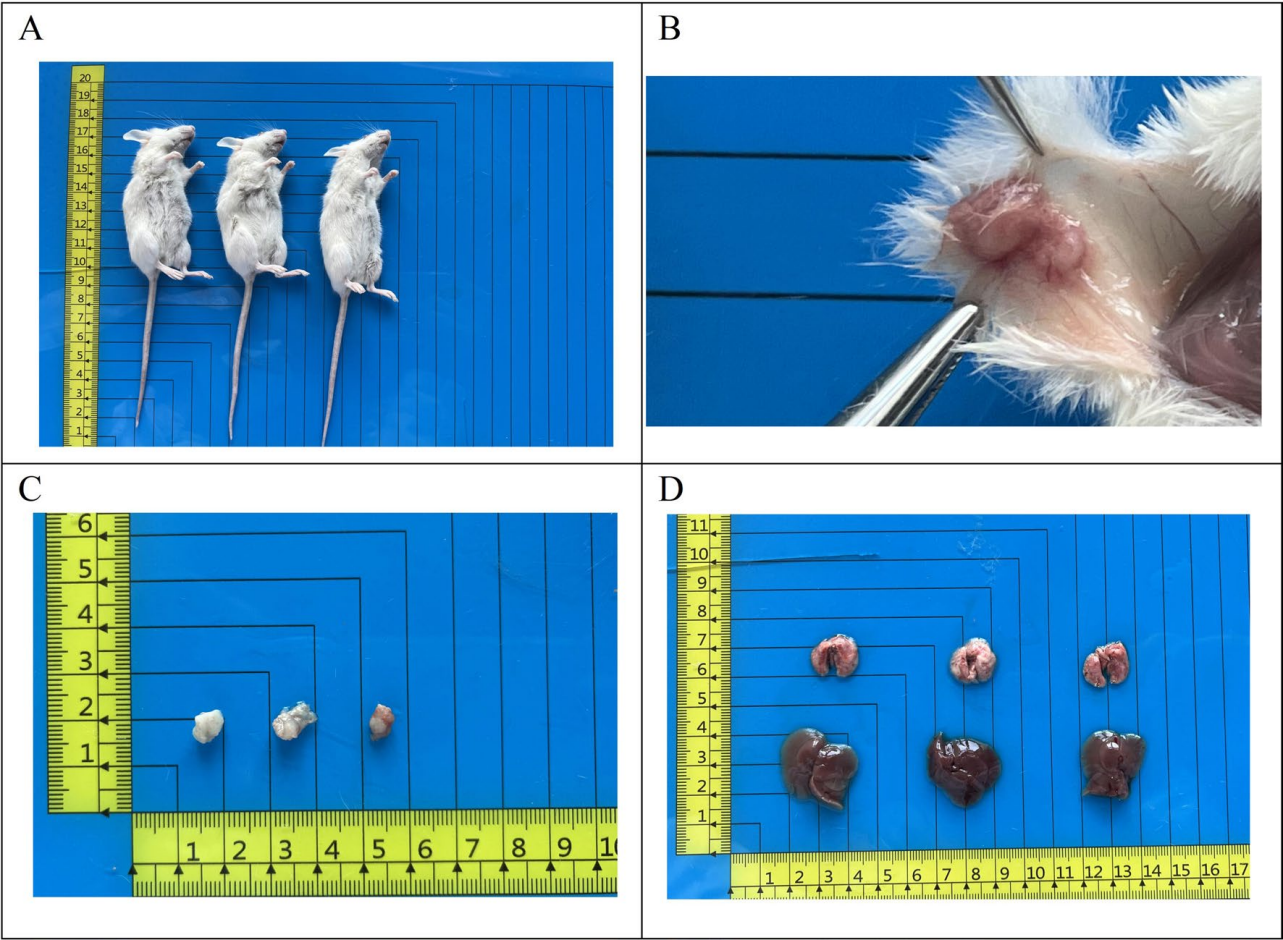
In vivo studies on xenograft formation by PDAC-X2 cells. (**A**–**C**) Subcutaneous transplantation of PDAC-X2 cells in NXG mice resulted in significant tumour formation (100% tumour formation rate). (**D**) No metastatic lesions were observed in the liver and lung tissues of nude mice at four weeks after transplantation.

Implantation of 1 × 10^6^ PDAC-X2 cells subcutaneously into three NXG mice resulted in 100% tumour formation within four weeks (Fig. 5A–C), confirming PDAC-X2’s ability to form transplanted tumours in vivo (100%) without observed metastatic lesions in the liver or lungs (Fig. 5D).

### H&E and immunohistochemical staining

**Figure 6:**
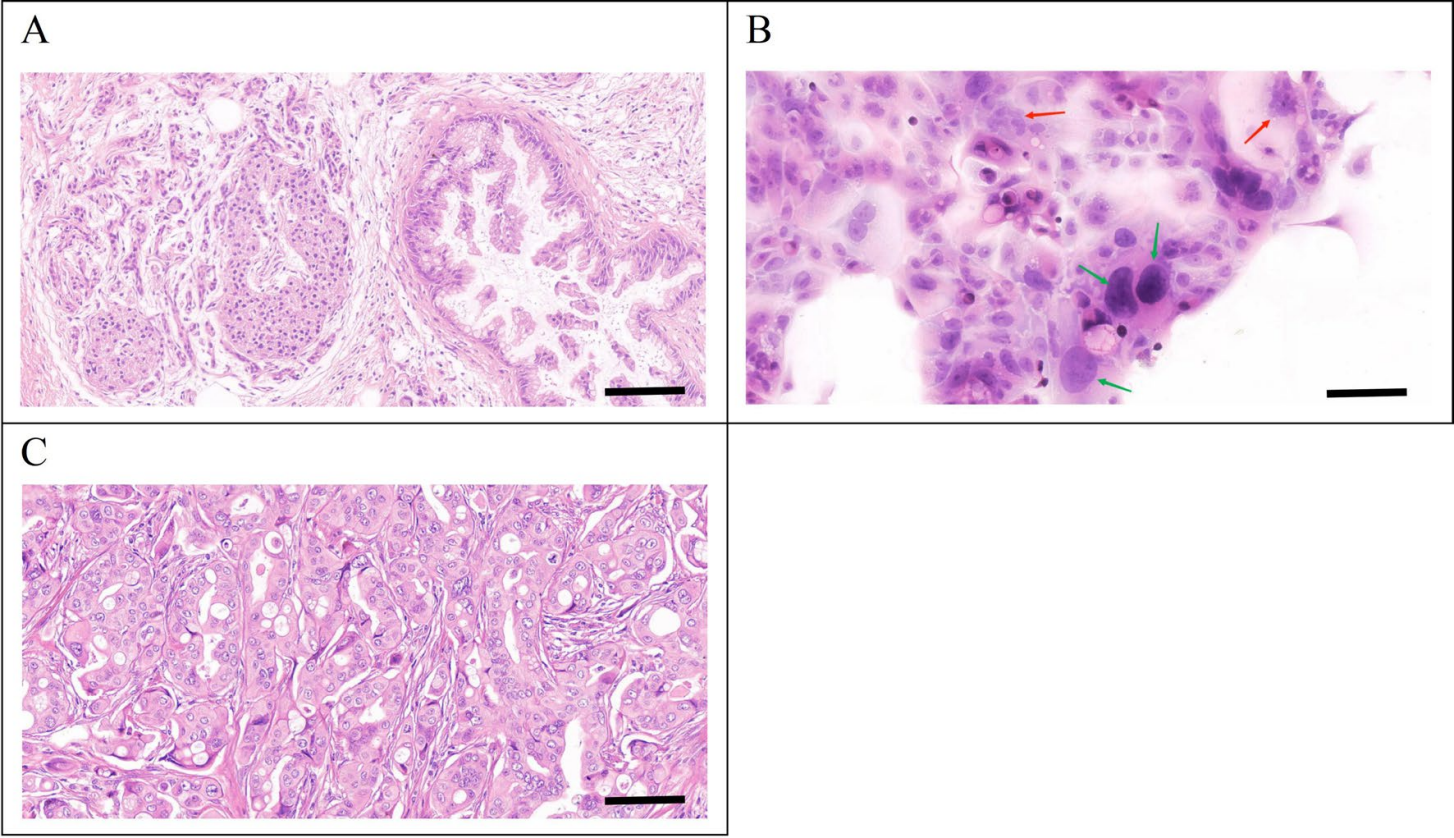
Hematoxylin–eosin (H&E) staining of the primary tumour, PDAC-X2 cells and xenografted tumour. The primary tumour showed moderately to poorly differentiated PDAC cells, with the tumour cells arranged in an irregular glandular tubular, cord-like and nest-like pattern, with abundant stromal components around the tumour (**A**). PDAC-X2 cells were significant differences in size. The nucleus was enlarged, the nucleolus was obvious and the cytoplasm was few. Multiple multinucleated (red arrow) and megakaryocytes (green arrow) were visible, presenting typical malignant tumour cell manifestations (**B**). The transplanted tumour showed irregular glandular-like cell structures, exhibiting moderate to low differentiation, and its histological morphology was similar to that of the primary tumour (**C**). (scale bar = 50 μm)

The postoperative pathological diagnosis of the primary tumour revealed moderately to poorly differentiated PDAC with irregular glandular, tubular, cord-like and nest-like patterns, surrounded by abundant stromal components (Fig. 6A).

H&E staining of PDAC-X2 cells displayed uneven morphology with pronounced variation in size, enlarged nuclei, prominent nucleoli and scant cytoplasm. Moreover, multiple multinucleated and megakaryocytes were visible, indicating typical malignant tumour cell manifestations (Fig. 6B).

H&E staining of the transplanted tumour showcased a clear boundary and was located within the subcutaneous fat layer, with local invasion into skeletal muscles. The arrangement of tumor cells was small nest like, glandular like, or trabecular like. The tumor cells were epithelioid, syncytial or columnar in shape, with abundant eosinophilic cytoplasm and some clear vacuoles visible in the cytoplasm; The nucleus was round or oval in shape, appearing as a bubble and of varying sizes. A single small nucleolus can be seen in the center, and nuclear division appears to be easily visible, resembling the histological morphology of the primary tumour (Fig. 6C).

**Figure 7:**
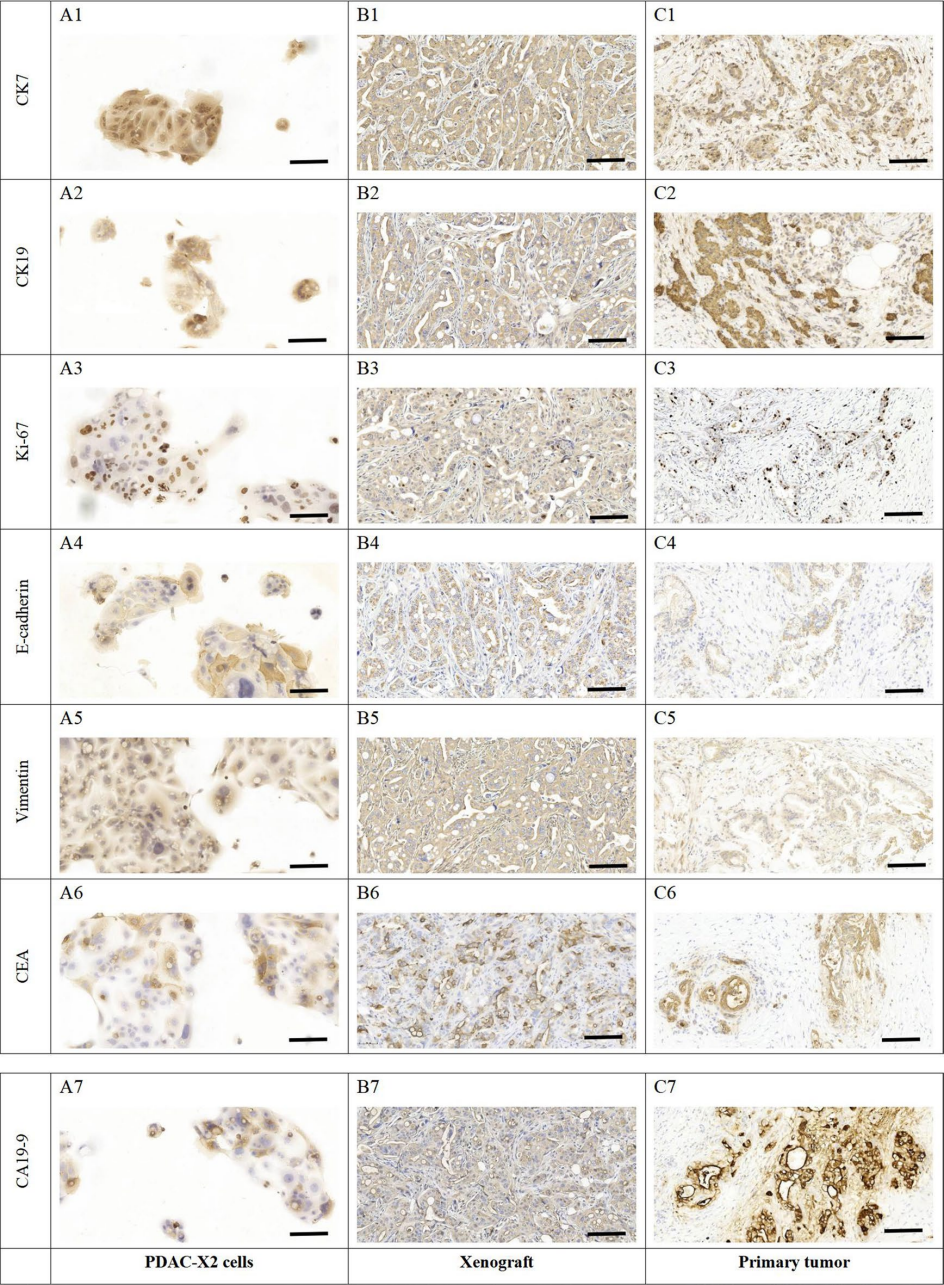
Immunohistochemical staining of PDAC-X2 cells, xenografted tumour and primary tumour. (**A1**), (**B1**) and (**C1**) CK7-positive staining in PDAC-X2 cells (**A1**), xenografted tumour (**B1**) and primary tumour (**C1**). (**A2**), (**B2**) and (**C2**): CK19-positive staining in PDAC-X2 cells (**A2**), xenografted tumour (**B2**) and primary tumour (**C2**). (**A3**), (**B3**) and (**C3**): Quantitative analysis showed a 40% positive rate of Ki-67 in PDAC-X2 cells (**A3**), xenografted tumour (**B3**) and primary tumour (**C3**). (**A4**), (**B4**) and (**C4**): E-cadherin-positive staining in PDAC-X2 cells (**A4**), xenografted tumour (**B4**) and primary tumour (**C4**). (**A5**), (**B5**) and (**C5**): Vimentin-positive staining in PDAC-X2 cells (**A5**), xenografted tumour (**B5**) and primary tumour (**C5**). (**A6**), (**B6**) and (**C6**): CEA-positive staining in PDAC-X2 cells (**A6**), xenografted tumour (**B6**) and primary tumour (**C6**). (**A7**), (**B7**) and (**C7**): CA19-9-positive staining in PDAC-X2 cells (**A7**), xenografted tumour (**B7**) and primary tumour (**C7**). (scale bar = 50 μm).

Immunohistochemistry exhibited positive expression of CK7 (Fig. 7A1–C1 and Supplementary Fig. 1A), CK19 (Fig. 7A2–C2 and Supplementary Fig. 1B), E-cadherin (Fig. 7A4–C4 and Supplementary Fig. 1D) and Vimentin (Fig. 7A5–C5 and Supplementary Fig. 1E) in cell lines, transplanted tumours and primary tumours. CEA (Fig. 7A6–C6 and Supplementary Fig. 1F) and CA19-9 (Fig. 7A7–C7 and Supplementary Fig. 1G) displayed weak positive expression, while Ki-67 (Fig. 7A3–C3 and Supplementary Fig. 1C) exhibited a 40% ratio, indicating rapid tumour proliferation.

## Discussion

Chemotherapy resistance in tumour cells poses a significant challenge in cancer treatment, obstructing therapeutic success. Therefore, exploring the underlying causes of drug resistance and strategies to overcome it remains a crucial and challenging research topic. Establishing chemotherapy-resistant cell lines serves as an important method for studying the mechanism underlying tumour cell resistance^21–23^. Here, we successfully established PDAC-X2, a novel pancreatic cancer cell line, derived from primary tumour tissue. We confirmed that PDAC-X2 cells exhibit multidrug resistance, characterising it as an intrinsic multidrug-resistant cell line. Thus, it serves as an excellent model for studying drug resistance mechanisms in pancreatic cancer.

Tumour heterogeneity, depicting molecular or genetic variations during tumour progression, influences distinct growth rates, invasive capabilities and drug sensitivities among tumour cells^24^. This heterogeneity, prevalent within large tumours, harbours different cell sets with distinct molecular characteristics and varying treatment sensitivities. Moreover, this heterogeneity could lead to uneven distribution of genetically different subpopulations of tumour cells between and within disease sites (spatial heterogeneity) or temporal changes in the molecular composition of cancer cells (temporal heterogeneity). Therefore, tumour heterogeneity is considered a primary cause of tumour treatment failure^25^. Pancreatic cancer is characterised by high heterogeneity between and within tumours, especially in terms of genetic changes and microenvironment. The drug resistance of pancreatic cancer patients to chemotherapy drugs is closely related to their heterogeneity^26,27^. Additionally, chromosomal instability and chromosomal aneuploidy are commonly present in human tumours^28,29^. Notably, the aneuploid karyotype of tumour cells is closely related to the poor prognosis of patients^30,31^. For instance, triploidy has also been associated with endogenous drug resistance in tumours, while tumours exceeding tetraploidy have been associated with acquired drug resistance^32^. PDAC-X2 cells have diverse morphologies and varying sizes, with multinucleated and megakaryocytes visible under light microscopy. The complex karyotype of PDAC-X2 indicates a high degree of heterogeneity, potentially driving multidrug resistance in PDAC-X2.

EMT, characterising the acquisition of mesenchymal features by epithelial cells, correlated cancer development, invasiveness, metastasis, and treatment resistance^33,34^. EMT involved decreased expression of epithelial genes, notably E-cadherin, and increased expression of mesenchymal genes like Vimentin. This transition leads to phenotypic changes, such as changes in cell morphology, loss of adhesion and acquisition of stem cell-like features. Crucial signalling pathways like transforming growth factor (TGF)- β), Wnt, Notch and hedgehog signalling pathways participate in EMT^35,36^. The decrease in E-cadherin expression and upregulation of Vimentin in PDAC-X2 cells indicate EMT characteristics, potentially contributing to its endogenous multidrug resistance.

Mouse tumour models serve as powerful tools for studying carcinogenesis, tumour progression and assessing therapeutic compound efficacy and toxicity. However, not all cell lines can form transplant tumours in nude mice^37,39^. In this study, PDAC-X2 was inoculated into NXG mice and exhibited the ability to rapidly form subcutaneous transplant tumours with a short incubation period and a 100% tumour formation rate. Pathological examination revealed a resemblance between PDAC-X2 transplanted tumours and primary tumours, emphasising its utility as a reliable in vivo experimental model.

In conclusion, the establishment of PDAC-X2, a novel human pancreatic cancer cell line from a Chinese female patient, presents a valuable addition to the repertoire of experimental models for fundamental research and novel drug development in pancreatic cancer.

### Supplementary Information

Supplementary Figure 1.

## Data Availability

The datasets used and/or analysed during the current study are available from the corresponding author upon reasonable request.
