# Supplementary material for: A novel multidrug-resistant cell line from a Chinese patient with pancreatic ductal adenocarcinoma
Source: Sci Rep. 2024 Apr 22;14:9259. doi: 10.1038/s41598-024-56464-w (PMC11035558; doi:10.1038/s41598-024-56464-w)
Supplement: Supplementary file 1 — Supplementary Figure 1. [file 41598_2024_56464_MOESM1_ESM.docx]

**Supplementary figure 1 The quantification of the stainings of PDAC-X2 cells, xenografted tumour and primary tumour.**

A: The quantification of the stainings of CK7-positive staining in PDAC-X2 cells, xenografted tumour and primary tumour .

B: The quantification of the stainings of CK19-positive staining in PDAC-X2 cells, xenografted tumour and primary tumour.

C: The quantification of the stainings of Ki67-positive staining in PDAC-X2 cells , xenografted tumour and primary tumour.

D: The quantification of the stainings of E-cadherin-positive staining in PDAC-X2 cells, xenografted tumour and primary tumour.

E: The quantification of the stainings of Vimentin-positive staining in PDAC-X2 cells, xenografted tumour and primary tumour.

F: The quantification of the stainings of CEA-positive staining in PDAC-X2 cells, xenografted tumour and primary tumour.

G: The quantification of the stainings of CA19-9-positive staining in PDAC-X2 cells, xenografted tumour and primary tumour.

The data are presented as the mean ± standard deviation (n = 3); *p < 0.05, **p < 0.01, ***p < 0.001,****p < 0.0001, ns=no statistical significance.

| 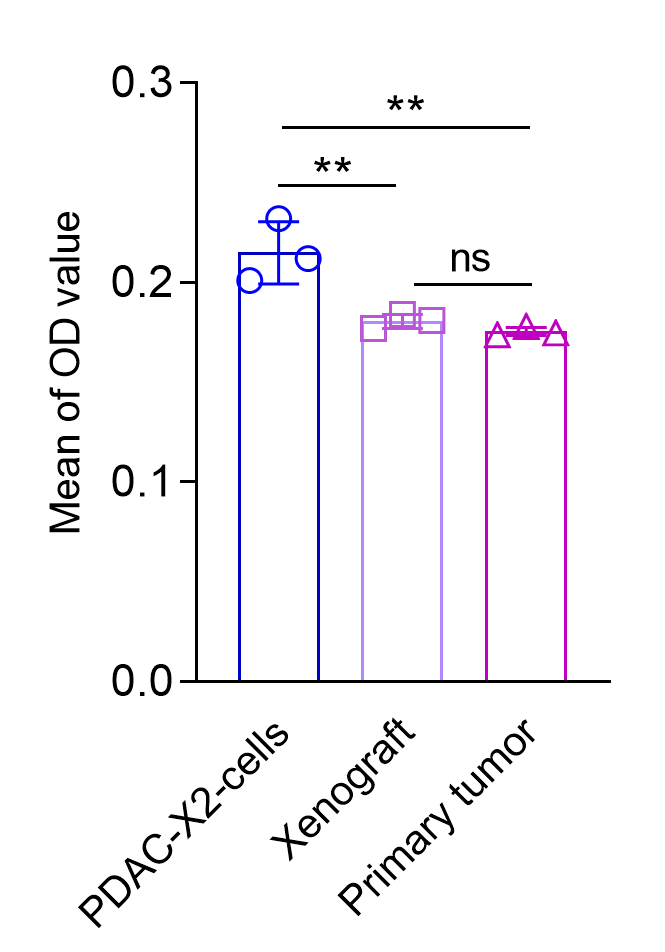A | 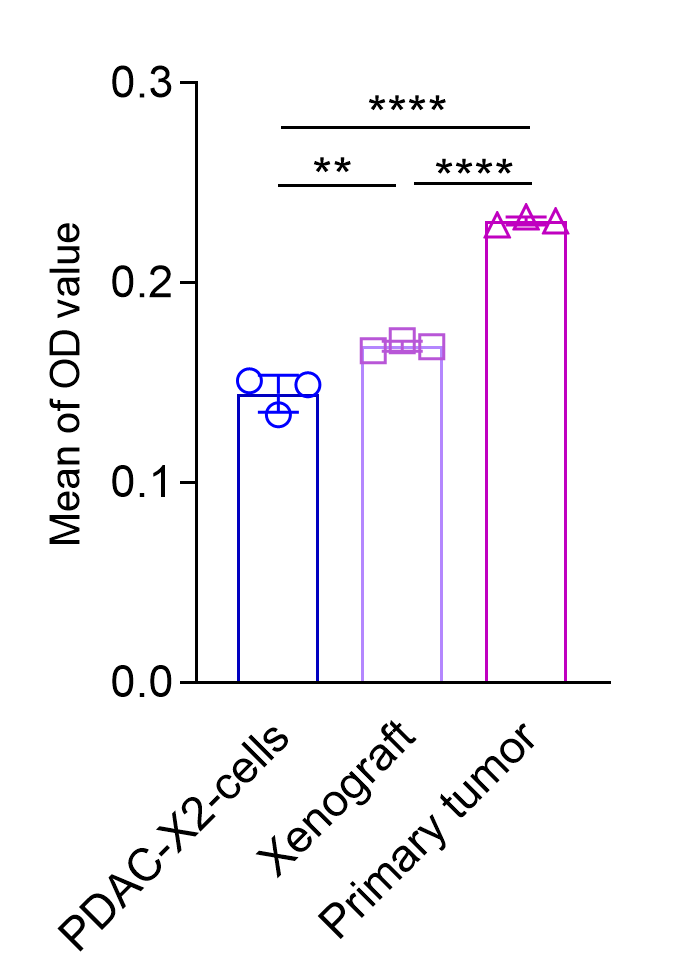B |
| --- | --- |
| 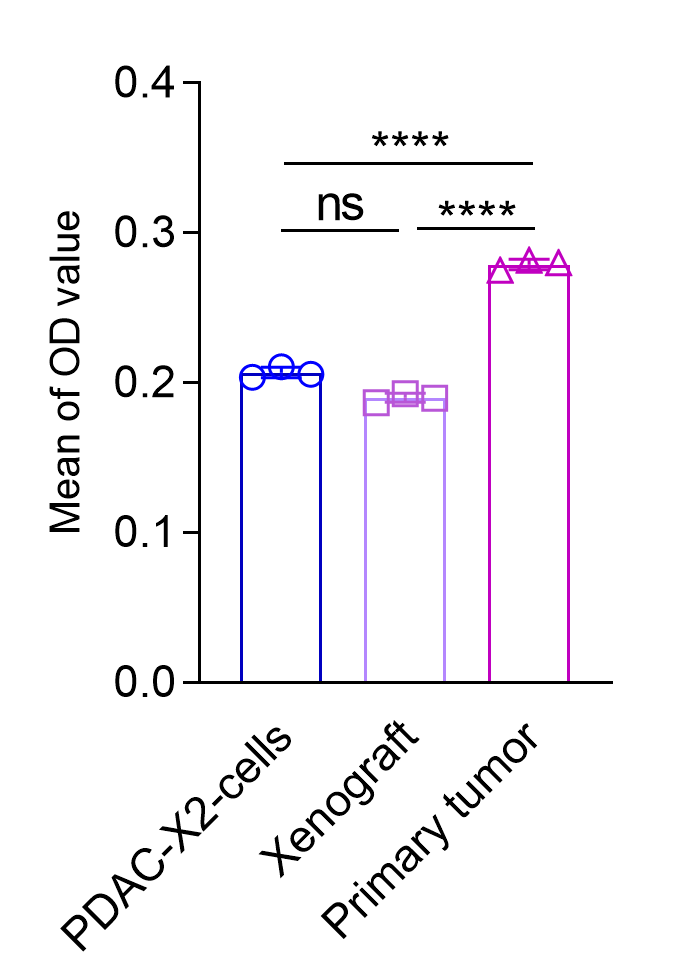C | 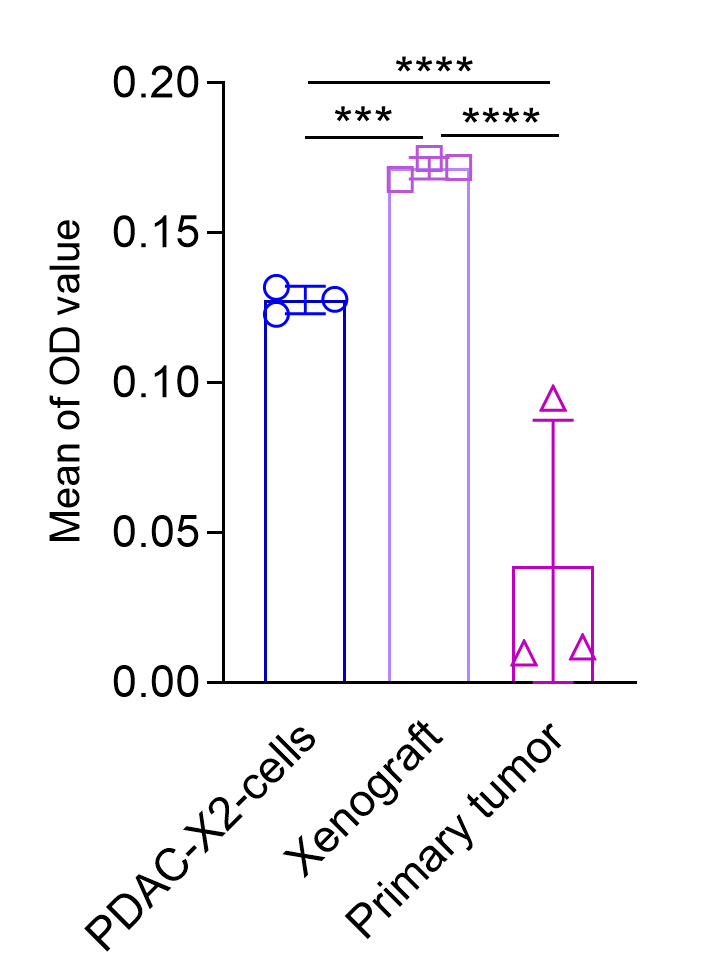D |
| 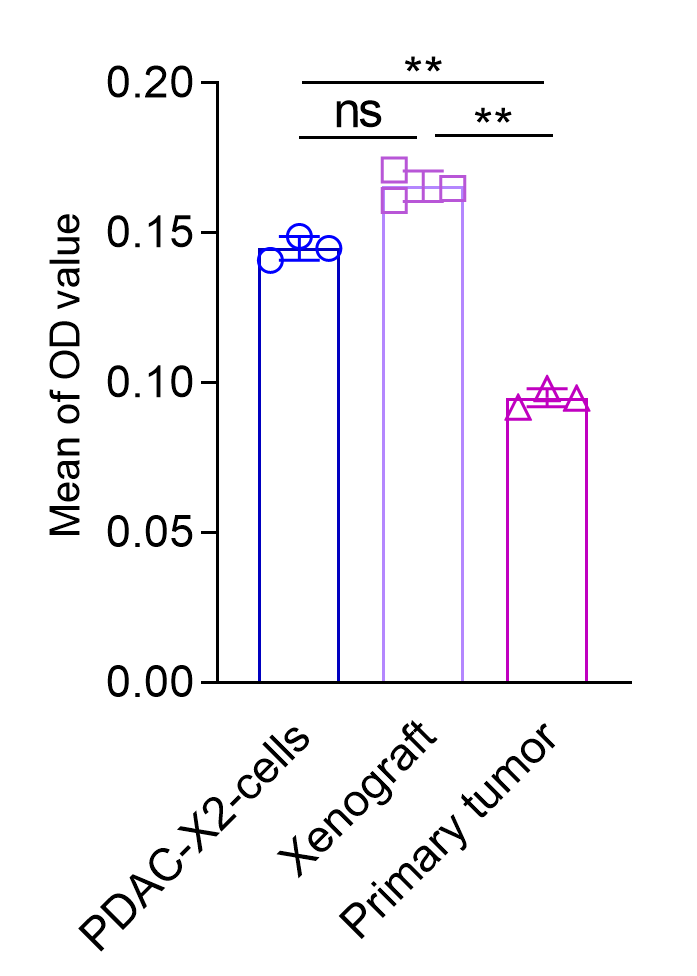E | 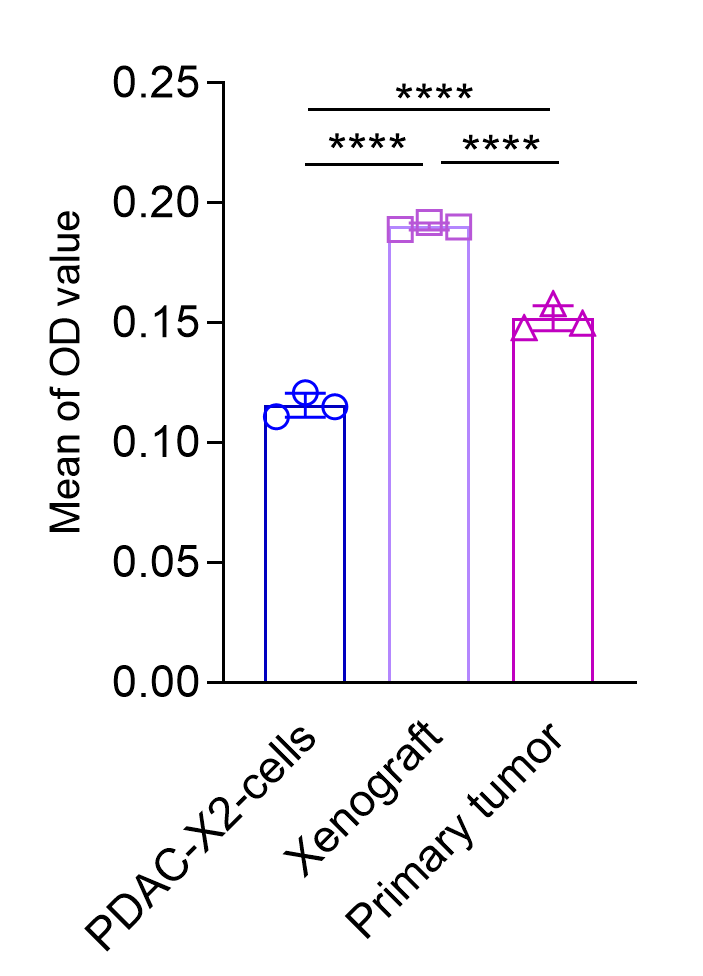F |
| 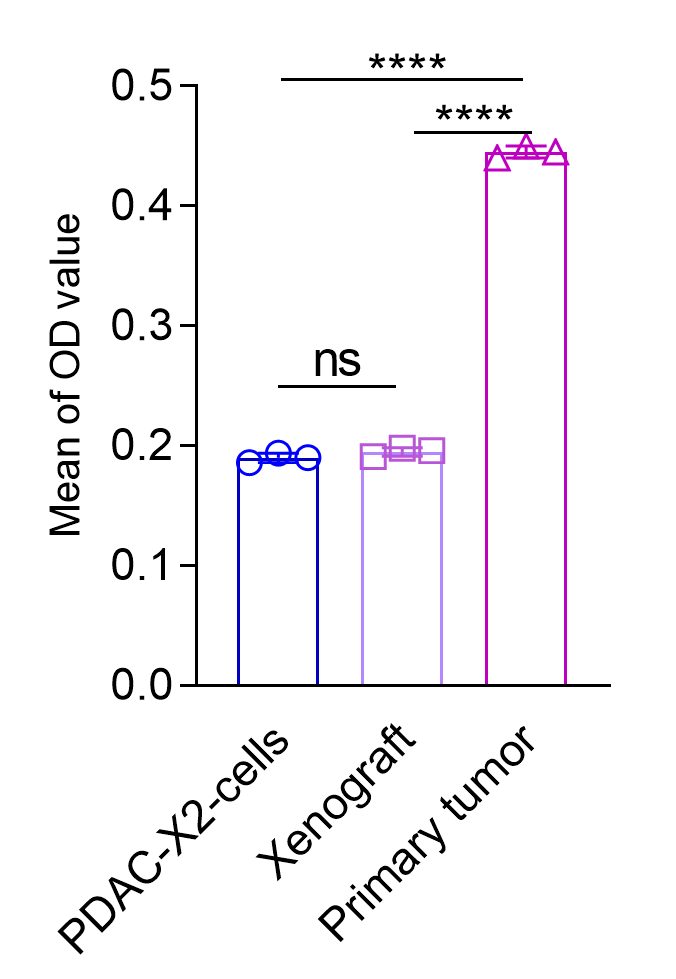G |  |
